# Statistical models for predicting the number of under-five mortality in Nepal

**DOI:** 10.1371/journal.pone.0324321

**Published:** 2025-05-21

**Authors:** Madhav Kumar Bhusal, Shankar Prasad Khanal

**Affiliations:** Central Department of Statistics, Tribhuvan University, Kirtipur, Kathmandu, Nepal; Group for Technical Assistance / Asian College for Advance Studies, Purbanchal University, NEPAL

## Abstract

**Background:**

Nepal has experienced the glacial reduction of under-five mortality in recent years which portends greater challenges to achieve the global target outlined in the Sustainable Development Goals (SDGs) for reducing childhood mortality. To resolve this inevitable adversity and safeguard newborns’ lives, additional scientific studies are necessary to plan for evidence-based interventions.

**Objective:**

This study aimed to develop a suitable statistical model using the associated factors to predict the number of under-five mortality a mother in Nepal encountered throughout her lifetime.

**Methods:**

The nationally representative Nepal Demographic and Health Survey (NDHS) 2022 data were used to conduct this study. The response variable was the number of under-five mortality a mother has experienced throughout her lifetime. Factors related to different circumstances, including maternal, paternal, socioeconomic, child, environmental, and the utilization of health care services were considered as independent variables. The zero-inflated negative binomial regression (ZINBR) model was fitted as the most plausible model to analyze the number of under-five mortality by its covariates after assessing the different count models including the Poisson regression (PR) model, negative binomial regression (NBR) model, zero-inflated negative binomial regression (ZINBR) model, and hurdle negative binomial regression (HNBR) model.

**Results:**

The analysis of the data revealed that 8.6% of the mothers in Nepal have endured at least one under-five mortality. The mean number of deaths before attaining five years was found to be 0.11 (95% CI: 0.10–0.11). The ecological region, smoking habit of mothers, total CEB, source of drinking water, preceding birth interval, and birth order number were obtained as significant covariates of the number of under-five mortality. Mothers from the mountain region (IRR: 1.1756, 95% CI: 1.0095–1.369, p-value = 0.0418) in contrast to hilly region, smoker mothers (IRR: 1.2067, 95% CI: 1.0392–1.4011, p-value = 0.0141) as compared to non-smoker mothers, mothers with total CEB 3 or more (IRR: 1.588, 95% CI: 1.3116–1.9226, p-value = 0.0353) as compared to 2 or less, preceding birth interval less than 24 months (IRR: 1.2061, 95% CI: 1.0788–1.3485, p-value = 0.0018) as compared to 24 months or more, and birth order number 4^th^ or above (IRR: 3.5681, 95% CI: 3.1512–4.0401, p-value < 0.001) as compared to 1^st^-3^rd^ were associated with the increased incidence of number of under-five mortality. Furthermore, the mothers who drink water from the tube well (IRR: 0.7719, 95% CI: 0.6191–0.9623, p-value = 0.0323) as compared to those who drink water from other sources have a decreased incidence of the number of under-five mortality.

**Conclusions:**

The ecological region, mother smokes cigarettes, total CEB, source of drinking water, preceding birth interval, and the birth order number appeared as the important predictors of under-five mortality. The high under-five mortality prevalent in the mountain and terai areas of Nepal presents a significant hurdle in efforts to decrease overall under-five mortality. Thus, improving existing healthcare services, raising the quality of life, and introducing effective health education initiatives are crucial to reducing under-five mortality.

## 1. Introduction

Under-five mortality rate (UFMR) refers to the likelihood of a newborn child dying before attaining the fifth birthday per 1000 live births. UFMR is one of the important indicators to assess the demographic characteristics of the country’s population, socioeconomic development, healthcare facilities, environmental conditions, and overall quality of life [1,2]. It is considered an outcome of the combination of nutritional status, immunization rates, maternal health, and other healthcare services received by mothers during pregnancy and after birth, and hence it is a marker to judge the health status of the community [3,4]. Moreover, UFMR is a reflector of a population’s well-being and a foundation for planning child and maternal health strategies [5].

The international community has continued to recognize under-five mortality as one of the disappointing facets of child health in the growing society in the Sustainable Development Goals (SDGs). This development initiative aims to address this issue by targeting a reduction in under-five mortality to 25 or fewer per 1,000 live births for all countries by 2030 [6]. The global under-five mortality declined by more than half during the Millennium Development Goals (MDGs) era (2000–2015) from 90 deaths per 1,000 live births in 1990 to 43 deaths per 1,000 live births in 2015. However, despite the significant progress achieved in reducing under-five mortality and improving maternal health, the tremendous efforts made were insufficient to meet the MDG objective of curbing under-five mortality by two-thirds between 1990 and 2015 [7]. The higher proportion of childhood mortality is still an incessant global public health issue, particularly in underdeveloped and developing countries. The widespread variations of high under-five mortality across different regions and levels require more concrete strategies for interventions that are relevant to the specific causes of death for its reduction [8]. Likewise, it is also suggested to enhance the availability and accessibility of preventive, promotive, and curative measures that lower mortality rates among children under five years, particularly in communities where the risk of child mortality is highest [9].

Globally, 4.9 million children lost their lives before their fifth birthday in 2022, and most of them are preventable loss of life. The unpleasant reality behind this fact is attributed to the possibility of child survival widely differs based on the place they are born, children born in low-income countries have a higher probability of dying before their fifth birthday, neonatal mortality occupies a higher proportion of under-five mortality, inequality in survival for children aged 1–59 months, children living in fragile and conflict-affected situations are particularly vulnerable, and communicable & infectious diseases are the leading sources of under-five deaths. Sub-Saharan Africa has the highest risk of UFMR in the world with 71 deaths per 1,000 live births, while the global UFMR recorded 37 per 1,000 live births in 2022. Similarly, Oceania (excluding Australia & New Zealand with only 4 deaths per 1,000 live births) is another region with a higher UFMR of 38 deaths per 1,000 live births, followed by Central & Southern Asia with 34 deaths per 1,000 live births [10].

In South Asia, the sluggish decline in neonatal mortality rates exacerbates the challenge of reducing under-five mortality, and it remains a major public health problem. The majority of under-five mortality is attributed to preventable and treatable causes like pre-term birth complications, intrapartum-related complications, and infections [11]. Out of the total under-five deaths recorded in 2022 in Central and Southern Asia, nearly 98 percent deaths accounted in the Southern Asia subregion. Among the 4.9 million under-five deaths reported globally in 2022, the Southern Asia subregion ascribed 1.25 million under-five deaths, which corresponds to 26 percent of the global total. The unusually high neonatal mortality is a burden on this region to diminish under-five mortality. Likewise, approximately 2 in 10 under-five deaths globally occur among children aged 1–59 months in Southern Asia [10]. A study on the varying age patterns of under-five mortality in this region revealed that under-five mortality in South Asia is marked by increased mortality rates among children younger than 28 days and those older than 6 months [12].

The global scenario regarding childhood mortality generally illustrates the inverse relationship between child mortality and the income level of the country. Despite this notion, Nepal is one of such low-income countries achieving a significant reduction in UFMR and stands out to prove that a paucity of economic prosperity is not a fence to save the lives of newborns [7]. In the past 25 years, Nepal has shown impressive strides in cutting child mortality rates. The UFMR dropped from 162 deaths per 1,000 live births in 1990–91 deaths per 1,000 live births in 2000, and this reduction further continued to fall to 38 deaths per 1,000 live births in 2014 [13]. Moreover, this reduction gained momentum, bringing down under-five deaths from 118 per 1,000 live births in 1996 to 33 per 1,000 live births in 2022, a reduction of 72 percent. Despite such continuous improvement in under-five mortality, neonatal mortality has remained constant since 2016. Likewise, the wide-ranging spatial variation in under-five mortality requires specific and effective interventions to further reduce such disparity and childhood mortality. The under-five mortality is highest in Sudurpashchim province (49 deaths per 1,000 live births), whereas the mortality was lowest in Gandaki province (23 deaths per 1,000 live births) in 2022 [1].

Nepal has made remarkable improvements in reducing under-five mortality in the MDG era. This achievement has resulted from the incessant implementation of periodic plans and strategies [14]. The substantial reduction in child mortality was possible due to the effective interventions to prevent or treat the major causes of childhood mortality through the various community-based and national campaign approaches. The foundation of this accomplishment was ascertained by the high financial investment by the government and other national and international non-government organizations (INGOs), United Nations (UN) agencies, and bilateral organizations. Likewise, the investment and initiatives made besides health sectors, primarily in women’s education, expansion of road network for vehicle movement, communication, improved drinking water, sanitation, construction of health facilities, and undernutrition have been taken as a national priority were the additional cornerstones for success. The other equally important drives were the strong political commitment to evidence-based health policy and partial progress in governance and leadership [14]. Despite the various initiatives and strong commitments made by the government and the continuous support of the international community, it seems to be challenging to further reduce neonatal mortality, which is almost unchanged in recent years, and under-five mortality to meet the global target of the SDGs.

Childhood mortality is a crucial subject of investigation for public health researchers in many countries around the globe. Few studies have been conducted in Nepal concerning neonatal, infant, and under-five mortality [15–23]. The majority of these studies on childhood mortality focused on neonatal and infant mortality [15–17,21–24]. These studies primarily utilized two statistical models: the multiple logistic regression model and hierarchical regression models to explore the factors associated with child survival/deaths. Some other studies concentrated on trend analysis and evaluating inequalities in neonatal mortality. They calculated the annual rate of reduction (ARR) and neonatal mortality rates using the Lorenz curve and a cohort log probability approach [18,22]. Similarly, two studies employed the Cox proportional hazards model to determine the factors linked to under-five mortality. One of these studies examined the trends over time and inequalities of under-five mortality in Nepal. This study used life table methods to calculate yearly under-five mortality from 1991 to 2010 according to the child’s sex, mother’s education, household wealth index, rural/urban residence, development regions, and ecological zones [20,24]. The aforementioned studies on childhood mortality in Nepal merely predict the status of survival/death of children rather than the number of survival/death of children a woman has seen in her lifetime. Studies on under-five mortality have applied the Cox proportional hazards model, and they revealed that children born to mothers who had previously lost a child, did not receive TT vaccinations, and did not use contraceptives faced a higher risk of dying before the age of five. Similarly, the mothers in the lowest quantile, compared to those in the highest, and mothers with no education, compared to those with higher education, were found to have a greater risk of under-five mortality [20,24]. The statistical models used for the analysis were limited to compare the hazard rates among different groups, while adjusting for confounders, and to determine the risk of death before the age of five. Furthermore, none of these studies have treated the outcome variable as a count variable characterized by overdispersion and excess zero values. These studies did not examine the number of child deaths a mother experiences throughout her lifetime or the factors associated with it.

Various models are strongly advised for forecasting the occurrence of under-five deaths based on their associated factors. Unlike the previous research conducted in Nepal, the outcome variable of this study consists of non-negative integers representing the frequency of discrete events, specifically defined as the total number of under-five deaths a mother has experienced in her lifetime. Statistical modeling of count data enables a more accurate and better estimation of the impact of policy interventions, either on the average occurrence rate or probability of no event, or the likelihood of one or multiple events [25]. The application of ordinary least squares (OLS) regression is unsuitable for the analysis of count data because these data do not meet the assumptions of normality and constant variance required by OLS regression. This can lead to unreliable estimates of standard errors, incorrect p-values, and misleading confidence intervals. It may generate a precise estimate of the dependent variable for one sample but fails to hold in other samples drawn from the same population [26,27]. In such situations, a more suitable approach to accurately address the unique features of count data is to utilize count data models. In light of these methodological advancements in count models, we have attempted to identify the significant covariates of the number of under-five mortality in Nepal by employing different count models, such as the Poisson regression (PR) model, negative binomial regression (NBR) model, zero-inflated negative binomial regression (ZINBR) model, and hurdle negative binomial regression (HNBR) model. Moreover, the PR is a basis for the analysis of count data. However, when there is excessive variability in the outcome variable, the model cannot adequately handle such overdispersed data, resulting in a significant underestimation of standard errors. One particular reason for overdispersion is the presence of zero inflation, which is greater than expected for the Poisson distribution. In this situation, one common choice is the ZINBR model. The ZINBR model is the preferred choice for effectively managing overdispersion and an excess of zero values [25,28]. As far as our current knowledge extends, no studies have attempted to predict the number of under-five mortality experienced by a woman throughout her lifetime using count data models suitable for the dataset exhibiting excess zeros and overdispersion within the context of Nepal. Hence, this study endeavors to investigate the factors and quantify their impact on the number of under-five mortality a woman has undergone in her lifetime, employing the Nepal Demographic and Health Survey (NDHS) 2022 data by an appropriate count model. The findings of this study are expected to provide meaningful insights to under-five mortality research by appropriately addressing overdispersion and excess zeros, and by offering deeper insights into high-burden families or communities, potentially guiding a shift in intervention strategies.

## 2. Methods

### 2.1 Data source and sampling procedure

We used one of the largest and most highly recognized NDHS 2022 datasets available in Nepal to assess the distribution of the number of under-five mortality. This dataset was obtained from the Demographic and Health Survey (DHS) program, ICF, Rockville, Maryland, USA, after submitting a brief description about the purpose of the study based on this data. The NDHS 2022 dataset incorporated up-to-date information on childhood mortality levels and other child, maternal, demographic, and health-related variables exclusively. The dataset encompassed a nationally representative sample of 14,280 households. Within these selected households, all women aged 15–49, or those within the same age group who remained overnight in the household preceding the survey, were eligible for participation in the study. The data were collected from January 5, 2022 and June 22, 2022.

As per the federal structure declared on September 15, 2015 by the Constitute Assembly of Nepal, an updated sampling frame was prepared to conduct the NDHS 2022 based on the frame used by the Central Bureau of Statistics (CBS) (now the office is named as National Statistics Office (NSO)) in the 2011 Nepal Population and Housing Census (NPHC). The NDHS 2022 adopted a two-stage stratified sampling technique to select the respondents. Altogether 14 sampling strata were created by dividing each of the seven provinces into rural and urban areas. In the initial stage, 476 primary sampling units (PSUs) were chosen. Among the selected PSUs, 248 were from urban areas and 228 were from rural areas. These PSUs were selected independently from each sampling stratum within the sample allocation and with probability proportional to PSU size. Before conducting the main survey, the household listing was finalized in all the selected PSUs. This list served as a sampling frame for the selection of households in the second stage. Moreover, in the cases of households listing when selected sub-wards were found excessively large with a projected number of households more than 300, then such sub-wards were segmented and just one segment was picked with the probability proportional to segment size for the survey. In the next step, 30 households were chosen from each cluster to get a total of 14,280 households in the sample. From the selected households, all women between the ages 15 and 49 years, whether they were permanent residents or visitors, were eligible to participate in the survey if they spent the night in the household just before the survey. These women were asked about numerous health-related issues, including their background characteristics, pregnancy history, year and month of each childbirth, and death. The complete procedure of data collection and sampling techniques used in the survey is described in the NDHS 2022 final report [1].

Altogether 14,845 women were interviewed in the survey. To develop a statistical model relating the under-five mortality to its covariates, respondents with missing values were eliminated. Therefore, the final models were fitted using the information from only 8119 respondents.

### 2.2 Study variables and data analysis

The response variable of this study was the total number of children who departed before reaching the age of 5 for each woman throughout her lifetime, and it is recorded in terms of counts as 0, 1, 2, 3, ……, n. The comprehensive risk factors associated with under-five mortality suggested by the literature and available in the NDHS 2022 dataset are included in the study [29]. These risk factors, their categories, and some summary statistics related to the number of under-five mortality are presented in Table 1. Most of the covariates were reclassified into new categories, while some retained their original categories as reported in the data. The levels of each covariate were defined as per the literature on childhood mortality, relevance to meaningful interpretation from a policy point of view, and considering the explorative analysis of this dataset. Some covariates had missing values. We did not use any imputation methods to replace the missing values. The cases with missing values were excluded from the analysis ensuring that the dataset has sufficient size for the study. The consistency of the data was confirmed by computing frequency tables and cross-tabulations. For data analysis, three different software programs were utilized. Data management tasks, including coding, recoding, and cleaning, were conducted in SPSS 26. Descriptive analysis, such as generating graphs for the outcome variable, calculating means, and determining standard deviations was also performed in SPSS 26. Additionally, stepwise regression analysis, incorporating both forward selection and backward elimination with an entry and removal probability of 0.05, was executed in STATA 18 using the “stepwise” command. Finally, all univariate and multivariate analyses were carried out in R (version 4.3.2) using the “MASS” and “pscl” packages.

### 2.3 Statistical models

Identical to other disciplines, in some studies related to public health and epidemiology, the response variable of interest consists of count data like the number of under-five mortality a woman has encountered during her lifetime. The statistical modeling of count data is analogous to the regression-type analyses provided that the outcome variable of interest is non-negative integers 0, 1, 2, etc. The count models are useful for estimating the effect of policy interventions either on the average rate or on the likelihood of no event, a single event, or two or more events [25]. The application of OLS regression for count data may not be appropriate due to the violation of normality and constant variance assumptions. The misspecification of the models can result in erroneous estimates of standard errors, leading to ambiguous p-values and confidence intervals [26]. In such a context, count models better analyze the outcome of interest from its covariates. These models enable us to evaluate how the count outcome changes as its covariates increase. In this study, we applied different statistical models, including PR, NBR, ZINBR, and HNBR models to investigate and measure the effect of various factors on the number of under-five mortality a mother experiences during her lifetime as per the nature of the data.

The count data models associated with the generalized linear models (GLMs) are developed on the foundation of Poisson distribution. PR is the most basic model for count data to determine the probability of count by Poisson distribution in which the mean of the distribution is a function of the independent variables. The inimitable feature of this model is that the conditional mean of the response variable is equal to the conditional variance. When conditional variance exceeds the conditional mean, it is a case of overdispersion. When the count data contain overdispersion of the outcome variable, then the NBR model is a better alternative to the PR model [30]. In the PR model if $y_{i}$ denote the number of under-five mortality for the i^th^ woman, and $\lambda_{i}$ denote the mean number of under-five mortality, then in the case when the number of under-five mortality follows Poisson distribution, its probability mass function is given by:


$$P\left(y_{i}|x_{i}\right)=\frac{e^{-\lambda_{i}}{\lambda_{i}}^{y_{i}}}{y_{i}!}\;,\;y_{i}=0,\;1,\;2,\;\ldots \ldots \ldots n,\;\lambda_{i}>0\;$$ (1)

The mean and variance of the Poisson distribution is $E\left(y_{i}\right)=Var\left(y_{i}\right)=\lambda_{i}$. If k denotes the number of independent variables, $x_{i^{\prime }s}$ are the covariates included in the model, and $\beta_{i^{\prime }s}$ are the corresponding coefficients, then the PR model using equation (1) can be expressed as:


$$\log\left(\lambda_{i}\right)=\beta_{0}+\beta_{1}x_{1}+\beta_{2}x_{2}+\ldots \ldots \ldots \ldots .+\beta_{k}x_{k}$$ (2)

The NBR model also referred to as negative binomial (NB) with two parameters, is constructed using the Poisson-gamma mixture distribution. The NBR model is recommended in the case of overdispersion or modeling the Poisson heterogeneity using a gamma distribution. This model provides the adjusted standard errors of regression coefficients and a more flexible method to predict the count response [31]. The probability distribution function of the NBR model for i^th^ observation is expressed as:


$$\mathrm{\backslash [}P\left(y_{i}|\lambda_{i},\alpha \right)=\frac{\Gamma \left(y_{i}+\alpha^{-1}\right)}{\Gamma \left(y_{i}+1\right)\Gamma \left(\alpha^{-1}\right)}{\left(\frac{1}{1+\alpha \lambda_{i}}\right)}^{\alpha^{-1}}{\left(\frac{\alpha \lambda_{i}}{1+\alpha \lambda_{i}}\right)}^{y_{i}},y_{i}=0,\;1,\;\ldots ;i=1,\;2,\;\ldots .n\mathrm{\backslash ]}$$ (3)

The NB distribution is characterized by two parameters, including a mean parameter $\lambda_{i}$, which denotes the mean number of under-five deaths per mother, and an overdispersion parameter α. The function Γ(.) is the gamma function. When α=0, the negative binomial distribution is the same as Poisson distribution, this means PR model is nested within the NBR model. The mean and variance are $E\left(y_{i}\right)=\;\lambda_{i}$ and $Var\left(y_{i}\right)=\lambda_{i}\left(1+\alpha \lambda_{i}\right)$.

Although the NBR model overcomes the issue of overdispersion in the PR model in count data analysis, it cannot adequately model over-dispersed count data caused by an excessive number of zeros. The appropriate model in such cases is to fit the data with a zero-inflated Poisson regression (ZIPR) model or ZINBR model [32]. The zero-inflated models inform about what predicts the occurrence and nonoccurrence of an event and what predicts the frequency of occurrences if the event occurs regarding the count outcome [33]. Moreover, these models account for the modeling of zero counts using both binary and count processes. These models assume two separate data generation processes for count data: the first process generates zeros only and the second data generating process is Poisson or negative binomial [31,34].

Moreover, the ZIPR model fails to fit the count data having excessive zeros with overdispersion. In such a case, the ZINBR is the recommended model. This model also consists of two distinct data generation processes, where $\omega_{i}$ is the probability for excess zeros and $\left(1-\omega_{i}\right)$ for the remaining counts that follow a negative binomial distribution [31]. If $Y_{i}$ denote the count of under-five deaths per mother, then the zero-inflated negative binomial (ZINB) distribution is given by:


$$\begin{array}{c} P\left(Y_{i}=y_{i}\right)=\left\{ \begin{array}{c} \omega_{i}+\left(1-\omega_{i}\right){\left(1+\alpha \mu_{i}\right)}^{-\frac{1}{\alpha }}\;,\;y_{i}=0\\ \left(1-\omega_{i}\right)\frac{\Gamma \left(y_{i}+\frac{1}{\alpha }\right)}{y_{i}!\Gamma \left(\frac{1}{\alpha }\right)}{\left(1+\alpha \mu_{i}\right)}^{-\frac{1}{\alpha }}{\left(1+\frac{1}{\alpha \mu_{i}}\right)}^{-y_{i}},\;y_{i}>0,\;0\leq \omega_{i}\leq 1 \end{array}\;\right.\; \end{array}$$ (4)

Where $\mu_{i}$ denote the mean of the negative binomial distribution. The over-dispersion parameter is α and it is independent of covariates. The mean of the ZINB distribution is with variance $Var\left(Y_{i}\right)=\left(1-\omega_{i}\mathrm{\backslash rightleft}(1+\omega_{i}\mu_{i}+\alpha \mu_{i}\right)\mu_{i}$. The ZINB distribution approaches to zero-inflated Poisson (ZIP) distribution when α→0. Likewise, the ZINB distribution converges to the negative binomial distribution as $\omega_{i}\to 0$. If α→∞ and $\omega_{i}\to 0$ the ZINB distribution reduces to the Poisson distribution [35].

In contrast to zero-inflated models, the hurdle models are two-part models. In these models, the first part is a binary response model and the second part is a zero-truncated count model [25]. The hurdle model assumes that all zero observations arise from structural sources only. The positive counts (i.e., non-zero) are due to sampling origin and follow either truncated Poisson or negative binomial distribution [36]. In the study of under-five mortality, it appears well to assume that only those women who did not have childbirth will possess zero under-five mortality during a given period, and the women who had given childbirth will have some positive (non-zero) number of under-five mortality during that period. Here the count zero can be obtained only from those women who did not have childbirth, originating from a structural source. If $Y_{i}$ denote the count of under-five deaths per mother, i = 1, 2, ……, n, where n is the total number of mothers, then the general structure of a hurdle model can be expressed as:


$$\begin{array}{c} P\left(Y_{i}=y_{i}\right)=\left\{ \begin{array}{c} \omega_{i}\;,\;y_{i}=0\\ \left(1-\omega_{i}\right)\frac{P\left(y_{i};\mu_{i}\right)}{1-P\left(y_{i}=0;\mu_{i}\right)}\;,\;y_{i}>0\; \end{array}\;\right.\; \end{array}$$ (5)

Where $\omega_{i}$ is the probability of mother belonging to the zero under-five mortality, $P\left(y_{i};\mu_{i}\right)$ denote the probability mass function for a positive count distribution with parameter $\mu_{i}$ and $P\left(y_{i}=0;\mu_{i}\right)$ is the distribution assessed at zero count.

Additionally, in the hurdle models, the hurdle negative binomial model is the most commonly used model for non-zero count when the Poisson distribution shows overdispersion [37,38]. The hurdle negative binomial model for the count response variable $Y_{i}$, (i = 1, 2, ……., n) is written as:


$$\begin{array}{c} P\left(Y_{i}=y_{i}\right)=\left\{ \begin{array}{c} \omega_{i}\;,\;y_{i}=0\\ \left(1-\omega_{i}\right)\frac{\Gamma \left(y_{i}+\frac{1}{\alpha }\right)\;{\left(1+\alpha \mu_{i}\right)}^{-\frac{1}{\alpha }}{\left(1+\frac{1}{\alpha \mu_{i}}\right)}^{-y_{i}}}{y_{i}!\Gamma \left(\frac{1}{\alpha }\right)\left(1-{\left(1+\alpha \mu_{i}\right)}^{-\frac{1}{\alpha }}\right)}\;,\;{\;y}_{i}>0 \end{array}\right.\; \end{array}$$ (6)

Where the dispersion parameter α>0, which is considered to be independent with covariates. Moreover, $0<\mu_{i}<1$ and $\omega_{i}=\omega_{i}\left(z_{i}\right)$. The logistic regression model used to model the probability of excess zero is given by:


$${logit\;(\omega }_{i})=log\left(\frac{\omega_{i}}{1-\omega_{i}}\right)=\sum_{j=1}^{q}z_{ij}\gamma_{j}\;$$ (7)

Where $Z_{i}=\left(1,\;z_{i1},z_{i2},\;\ldots \ldots ..,z_{iq}\right)$ is the i^th^ row of covariate matrix *Z*. $\gamma =\left(\gamma_{1},\gamma_{2},\;\ldots \ldots \ldots ,\gamma_{q}\right)$ are unknown q-dimensional column vectors of parameters. The influence of covariates on count outcomes using NBR can be modeled as:


$${log(\mu }_{i})=\;\sum_{j=1}^{p}x_{ij}\beta_{j}$$ (8)

Where $x_{{ij}^{\prime }s}$ are the covariates, $\beta_{j^{\prime }s}$ are the regression coefficients, and p denotes the number of covariates.

**Fig 1:**
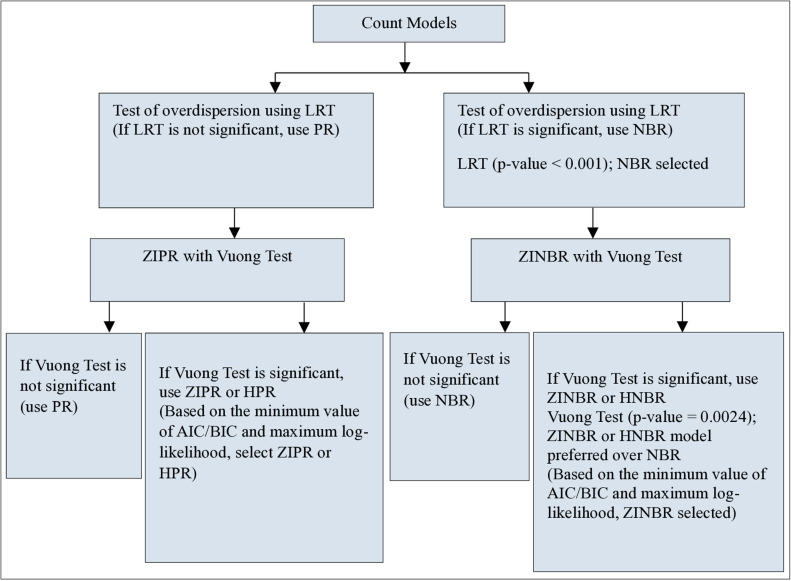
Flowchart for the selection of count regression model to analyze the under-five deaths in Nepal.


Fig 1 illustrates a visual representation of the model selection process, developed using insights from the prior study [39]. The presence of overdispersion in the number of under-five deaths was assessed using the likelihood ratio test (LRT). The LRT evaluates the nested models (i.e., NBR vs. PR, ZINBR vs. ZIPR, and HNBR vs. HPR) and confirms the necessity of the overdispersion parameter. This study employs LRT to compare two nested models, PR versus ZINBR to account for overdispersion. As mentioned earlier, the Poisson model is a special case of the negative binomial model with a dispersion parameter α=0. The statistical hypotheses for the LRT to test overdispersion are defined as: the null hypothesis (H_0_): α=0 (no overdispersion) against the alternative hypothesis (H_1_): α>0 (overdispersion). The LRT statistic is expressed as;

LRT statistic= −2(Log−likelihood of Poisson model−Log−likelihood of Negative Binomial model         (9)

The LRT statistic follows the Chi-square distribution with a degree of freedom equal to the difference in the number of parameters in the model. The significant result of LRT indicates the presence of overdispersion. In this situation, negative binomial models are more appropriate than the Poisson models as shown in (Fig 1) [25,38]. Further, the Vuong test was used to confirm the suitability between pairs of non-nested models, particularly to compare the zero-inflated count models with their standard analogs. In this study, it is used to compare ZINBR versus NBR. The null hypothesis under the test assumes that the two models NBR and ZINBR are indistinguishable versus the alternative hypothesis, the ZINBR is better than the NBR model. The Vuong test statistic is asymptotically distributed as a standard normal variate [40]. The significant result of the Vuong test suggests that either the ZINBR or HNBR model is suitable for predicting the number of under-five mortality. Finally, the ZINBR and HNBR models were compared using the maximum log-likelihood value and the lowest Akaike information criterion (AIC) and Bayesian information criterion (BIC) values. The overall goodness fit of the most plausible model was assessed using the Pearson Chi-square test statistic [41].

In our study, the ZINBR model emerged as the most appropriate count model for evaluating the factors influencing under-five mortality. Multiple factors may render the ZINBR model more appropriate than the HNBR model for explaining under-five mortality. The zero-inflated models assume that excess zeros (no under-five mortality) result from two distinct processes. One process identifies the presence of excess zeros resulting from individuals having strong protective factors such as adequate nutrition, access to healthcare facilities, and favorable socioeconomic conditions, while the other process models the actual count of under-five mortality among those at risk. The results of this study support this notion, as under-five mortality exhibited disparities across different groups and circumstances. However, in the HNBR model, all zero outcomes are attributed solely to the binary process, assuming that zero under-five mortality cannot occur from the count component of the model. In other words, the ZINBR model accounts for both structural zeros (cases where there is no risk of under-five mortality) and sampling zeros (cases where children are at risk but do not experience under-five mortality). In contrast, the HNBR model assumes that if a child is at risk, the absence of under-five mortality cannot occur by chance. This implies that the ZINBR offers a more suitable framework for managing these excess zeros, whereas the hurdle model assumes all children are at some risk, which may not accurately reflect reality.

The incidence rate ratio (IRR) in the count part of the ZINBR model is computed based on the association between the expected under-five mortality count and the linear predictors of the model. In the count part of ZINBR model, the outcome variable is assumed to follow a negative binomial distribution characterized by a mean µ and dispersion parameter α. The expected mean count is $\mu =e^{X\beta }$. Here X denotes the matrix of predictors for the count part and *β* denotes the vector of regression coefficients. The IRR quantifies the multiplicative change in the expected count associated with a one-unit increase in the predictor $X_{j}$, holding all other predictors constant. For a particular predictor $X_{j}$, it’s coefficient $\beta_{j}$ determines the IRR by using the relation ${IRR}_{j}=e^{\beta_{j}}$.

Similarly, the adjusted odds ratio (AOR) in the zero-inflated part of the ZINBR model is derived from the coefficients of the zero-inflation model, which uses a binomial distribution with a logit link function. In this model, the dependent variable is modeled as a binary outcome. The probability of observing the structural zero is determined using a logistic regression, expressed as;


$$\omega_{i}=\frac{e^{Z\gamma }}{1+e^{Z\gamma }}$$

Where $\omega_{i}$ is the probability of structural zero, *Z* denotes the matrix of predictors for the zero-inflation part, and γ denotes the vector of regression coefficients of the model [31].

For a given predictor $Z_{j}$, the odds of being a structural zero can be written as


$$Odds=\;\frac{\omega_{i}}{1-\omega_{i}}=\;e^{Z\gamma }$$

The AOR for a particular predictor $Z_{j}$ can be obtained as


$${AOR}_{j}=e^{\gamma_{j}}$$

### 2.4 Ethical approval

This research used the secondary data accessed from the Demographic and Health Survey (DHS) program. The NDHS 2022 data collection procedures were approved by ICF Macro International (Calverton, Maryland, USA) Institutional Review Board and the survey protocol was reviewed by the Nepal Health Research Council (NHRC). Written consent was obtained from the household head to conduct the interviews.

## 3. Results

### 3.1 Descriptive analyses

The analysis of NDHS 2022 data after accounting for the sampling weights revealed that 14,821 women aged 15–49 were included in this study. Among them, most of the mothers 13,541 (91.4%) had no under-five mortality during their lifetime. Of those involved in the survey 1,280 (8.6%) mothers had one up to six under-five mortality in their lifetime. Among the total 1,055 (7.1%) mothers experienced one under-five mortality followed by 167 (1.1%) mothers with two under-five mortality in their lifetime.

**Fig 2:**
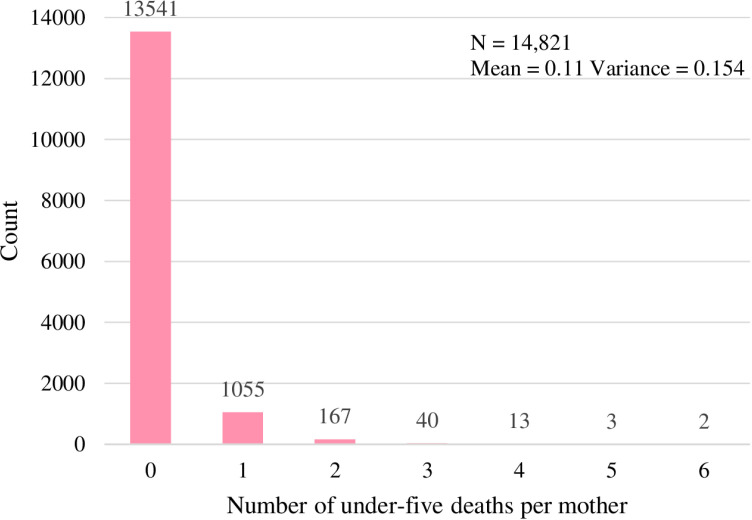
Distribution of the number of under-five deaths experienced by a mother during her lifetime.

Fig 2 illustrates that the average number of under-five mortality a mother had encountered over her life was 0.11, with a 95% confidence interval of (0.10, 0.11), and a variance of 0.154. The variance greater than the mean indicates the possibility of overdispersion. Moreover, the number of under-five mortality demonstrates the right-skewed distribution with excess zero observations. Zero-inflated models or hurdle models might be appropriate count models to better explain such data structure.

Table 1 shows that the average number of under-five mortality was highest in the mountain region (0.18) followed by terai (mean = 0.11), and the lowest was reported in the hill region (mean = 0.09). The Karnali province recorded the highest anticipated number of under-five mortality (mean = 0.18), followed by Sudurpaschim (mean = 0.15), Madhesh province (mean = 0.14), and Lumbini province (mean = 0.11), whereas the lowest was reported in Bagmati and Gandaki province (mean = 0.07). Likewise, concerning the place of residence, rural areas showed a higher mean number of under-five mortality (mean = 0.15) as compared to urban areas (mean = 0.09). The anticipated number of under-five deaths exhibits an inverse correlation with the educational attainment of both mothers and fathers of children. Mothers with no education reported a significantly higher mean number of under-five mortality (mean = 0.27), whereas the lowest was reported for those mothers achieving secondary and higher education (mean = 0.02).

**Table 1:** Descriptive statistics of under-five mortality during the lifetime of a mother by various circumstances, NDHS 2022.

Variables	Categories	n	Percent	Mean	Std. deviation
Ecological region	Mountain	790	5.33	0.18	0.516
	Hill	5865	39.57	0.09	0.386
	Terai	8166	55.10	0.11	0.381
Province	Koshi	2492	16.82	0.08	0.331
	Madhesh	3001	20.25	0.14	0.41
	Bagmati	3060	20.65	0.07	0.322
	Gandaki	1399	9.44	0.07	0.36
	Lumbini	2683	18.10	0.11	0.41
	Karnali	907	6.12	0.18	0.451
	Sudurpashchim	1278	8.62	0.15	0.451
Place of residence	Urban	10167	68.60	0.09	0.357
	Rural	4654	31.40	0.15	0.392
Education level of mother	No education	3781	25.51	0.27	0.609
	Basic	4586	30.94	0.09	0.348
	Secondary	5798	39.12	0.02	0.165
	Higher	656	4.43	0.02	0.131
Education level of husband	No education	1475	13.41	0.27	0.589
	Basic	4458	40.52	0.15	0.456
	Secondary	4247	38.61	0.07	0.304
	Higher	821	7.46	0.06	0.307
Source of drinking water	Piped water	7070	47.70	0.11	0.405
	Tube-well	5252	35.44	0.13	0.408
	Others	2499	16.86	0.06	0.305
Time to get water source	Outside premises	1999	14.29	0.16	0.489
	On premises	11989	85.71	0.1	0.381
Type of toilet facility	No facility/bush/field	872	5.88	0.19	0.507
	With facility (any type)	13948	94.12	0.1	0.383
Household access to electricity	No	433	3.09	0.17	0.481
	Yes	13571	96.91	0.11	0.396
Main floor/roof/wall material of house	Finished	7208	51.47	0.07	0.327
	Others	6796	48.53	0.15	0.46
Religion of mother	Hindu	12355	83.36	0.11	0.385
	Others	2466	16.64	0.11	0.424
Ethnicity of mother	Brahmin/Chhetri	4329	29.24	0.09	0.369
	Other terai caste	2147	14.50	0.14	0.406
	Dalit	2237	15.11	0.14	0.466
	Janajati	5417	36.59	0.09	0.361
	Muslim	674	4.55	0.13	0.44
No. of household members/Family size	4 or less	6944	46.86	0.1	0.39
	5 or more	7876	53.14	0.11	0.393
No. of children age 5 or below in household	No children	8680	58.57	0.11	0.392
	1-2	5775	38.97	0.11	0.391
	3 or more	365	2.46	0.13	0.386
Sex of household head	Male	9729	65.65	0.11	0.395
	Female	5091	34.35	0.11	0.385
Access to mass media	No access	3129	21.11	0.16	0.476
	Access	11692	78.89	0.09	0.365
Type of cooking fuel	LPG	5912	39.89	0.06	0.301
	Wood	6859	46.28	0.14	0.447
	Others	2049	13.83	0.12	0.412
Wealth index	Poorest	2622	17.69	0.19	0.518
	Poorer	2847	19.21	0.14	0.444
	Middle	3023	20.40	0.11	0.376
	Richer	3194	21.55	0.08	0.345
	Richest	3134	21.15	0.04	0.23
Total CEB	2 or less	10602	71.53	0.02	0.132
	3 or more	4219	28.47	0.33	0.651
Mother smokes cigarettes	No	14256	96.19	0.1	0.376
	Yes	564	3.81	0.3	0.65
Travel time to nearest healthcare facility (min.)	30 or less	13199	89.06	0.1	0.372
	More than 30	1622	10.94	0.17	0.518
Husband’s working status	Not working	287	2.59	0.16	0.463
	Professional/technical/managerial	1238	11.16	0.08	0.338
	Agriculture	2051	18.50	0.18	0.504
	Skilled/unskilled manual	4940	44.55	0.14	0.435
	Others	2573	23.20	0.09	0.362
Mother’s working status	Not working	4146	27.97	0.304	0.304
	Professional/technical/managerial	858	5.79	0.216	0.216
	Agriculture	7113	47.99	0.456	0.456
	Skilled/unskilled manual	1230	8.30	0.41	0.41
	Others	1474	9.95	0.301	0.301
Birth order number	1^st^-3^rd^	8521	80.63	0.06	0.249
	4^th^ or more	2047	19.37	0.53	0.802
Type of birth	Single	10464	99.01	0.15	0.455
	Multiple	105	0.99	0.29	0.603
Preceding birth interval	Less than 24 months	1542	19.57	0.29	0.638
	24 months or more	6337	80.43	0.18	0.48
Number of TT injections received by mother before childbirth	Less than 2	1090	39.75	0.15	0.44
	2 or more	1652	60.25	0.07	0.307
Delivery assistance	Traditional birth attendant	198	8.2	0.17	0.448
	Health professionals	2225	91.8	0.07	0.293
Number of ANC visits	4 or more	2262	81.81	0.09	0.329
	Less than 4	503	18.19	0.17	0.499
Place of delivery	Health centers (any)	2236	80.87	0.08	0.305
	Home	529	19.13	0.22	0.545
Mode of delivery	Caesarean section	549	19.86	0.05	0.233
	Normal	2216	80.14	0.12	0.392
Size of child at birth	Very large	55	2.01	0.17	0.416
	Larger than average	286	10.45	0.14	0.395
	Average	2002	73.17	0.09	0.342
	Smaller than average	272	9.94	0.12	0.486
	Very small	121	4.42	0.14	0.377
PNC within first two days after birth	No	1889	69.60	0.11	0.39
	Yes	825	30.40	0.08	0.309
Age of mother at childbirth	Below 20	1086	10.27	0.04	0.226
	20-25	3866	36.58	0.09	0.324
	25-30	3462	32.76	0.16	0.474
	30-35	1621	15.34	0.27	0.599
	35 or more	533	5.04	0.35	0.764
Child received all basic antigen	No (none)	179	0.098	0.09	0.358
	Yes	1645	0.902	0.09	0.348
Child fully vaccinated	No (partial)	2111	75.3	0.10	0.359
	Yes	694	24.7	0.07	0.303

Considering the time to get the water source, the household with the water facility outside the premises had a higher number of under-five deaths (mean = 0.16), whereas the lower expected number (mean = 0.1) was observed in the household having water facility within the premises. Similarly, the higher anticipated count of under-five mortality (mean = 0.19) was recorded in the households without toilet facility than in the households with any type of toilet facility (mean = 0.1). It is also found that households with no access to electricity (mean = 0.17) and having an unfinished type of house (mean = 0.15) experience a higher average number of under-five deaths. The expected number of under-five deaths was found identical among the respondents belonging to the Hindu faith and adhering to other religions. Regarding the mother’s religious affiliation, Table 1 revealed that the average under-five mortality was elevated among the mothers from Dalit, Muslim, and other Terai castes, whereas it was lowest among Brahmin/Chhetri and Janajati mothers. Furthermore, the households having 3 or more children aged 5 or below had high under-five deaths (mean = 0.13), in contrast, the households with 1–2 or no children aged 5 or below experienced a lower mean number of under-five mortality (mean = 0.11). Mothers who had access to mass media experienced a lower expected number of under-five mortality (mean = 0.09) meanwhile, a greater average number of under-five mortality was observed among those who had no access to mass media (mean = 0.16). Similarly, households that utilize wood for cooking exhibited a higher mean number of under-five deaths (mean = 0.14) compared to those using LPG for cooking (mean = 0.06). Table 1 also shows an inverse correlation between households’ wealth index and the expected number of under-five mortality. The households categorized under the poorest wealth index encountered the highest under-five mortality (mean = 0.19), whereas the lowest mortality was observed in those households belonging to the richest wealth index (mean = 0.04). On the other hand, mothers with 3 or more CEB were found to have a higher expected number of under-five deaths (mean = 0.33) than those mothers with 2 or fewer CEB (mean = 0.02).

Mothers who smoke cigarettes experienced a higher mean number of under-five mortality (mean = 0.3) than those who did not smoke. Likewise, mothers who traveled for more than 30 minutes from their house to the nearest healthcare facility exhibited the highest average number of under-five mortality (mean = 0.17) compared to those who traveled 30 minutes or less. The average number of under-five deaths was highest among those mothers where the primary occupation of both husband and wife was agriculture (mean = 0.18) and (mean = 0.456) respectively. It was also found that the mean number of under-five mortality is positively correlated with birth order number. The expected number of under-five mortality was highest (mean = 0.53) for those mothers with birth order 4 or more as compared to those with 1^st^-3^rd^ birth order. The expected number of under-five mortality was also found to differ according to types of birth whether it was single or multiple. The average number of under-five mortality was highest in multiple births (mean = 0.29), while for single births the mortality was (mean = 0.15). Furthermore, the highest mean number of under-five mortality was reported for a preceding birth interval less than 24 months (mean = 0.29) and the lowest was reported for a preceding birth interval 24 months or above (mean = 0.18) suggesting that the preceding birth interval was inversely associated with the under-five mortality. The mean count of under-five mortality was reported lowest for those mothers who received 2 or more TT injections before childbirth (mean = 0.07) whereas the highest mortality (mean = 0.15) was observed for those mothers who received less than 2 TT injections. Mothers who received delivery assistance from traditional birth attendants exhibited the highest expected number of under-five mortality (mean = 0.18) compared to those assisted by health professionals. It was also found that the average number of under-five mortality was greater among individuals who underwent home deliveries (mean = 0.22) than those mothers who gave birth at healthcare centers. Similarly, mothers who visited 4 or more times for ANC detected the lowest mean number of under-five mortality (mean = 0.09) as compared to those who had less than 4 ANC visits. Additionally, the mothers who had a normal delivery (mean = 0.12) and did not receive postnatal care within the first two days after childbirth had the highest expected number of under-five mortalities. Furthermore, there is a positive correlation between the age of the mother at childbirth and the mean number of under-five mortality. The highest average number of under-five mortality was observed among mothers aged 35 and above, followed by those in the age group of 30–35. There was no noticeable difference in the expected number of under-five mortality between children who received basic antigen vaccines and those who did not. Nevertheless, the lowest mean number of under-five mortality was recorded among children who were fully vaccinated compared to those who were not fully vaccinated.

### 3.2 Inferential analysis

#### 3.2.1 Test of over-dispersion and variables selection for model fit.

Fig 2 exhibits that the variance of the under-five mortality surpasses the mean. To assess this result, test for the overdispersion (α) was executed. This test aimed to decide the null hypothesis of no overdispersion against the alternative hypothesis that there exists overdispersion in the outcome variable. The likelihood ratio test was performed to examine the hypothesis, and it provided a likelihood ratio test statistic of 29.575 with a p-value of less than 0.001. This result indicates the evidence of the presence of overdispersion and implies the need for the NBR model to fit the data.

To explore the significant covariates of under-five mortality out of various covariates as shown in Table 1, the bivariate analysis was performed by taking the outcome variable and each covariate separately for each of the PR and NBR. By this analysis, both PR and NBR excluded seven variables- religion of mother, number of household members, number of children age 5 or below in house, sex of household head, PNC within two days after birth, child received all basic antigen, and whether the child was fully vaccinated or not out of 36 covariates included in the analysis at 0.05 level of significance. Moreover, the forward and backward stepwise regression methods were employed for the further selection of covariates by considering 29 variables as the candidates for the model. Both methods selected the same set of seven covariates. The selected variables were ecological region, place of residence, mother smokes cigarettes, total CEB, source of drinking water, preceding birth interval, and birth order number at entry and removal probability 0.05.

#### 3.2.2 Model selection to predict the number of under-five mortality.

The presence of overdispersion abolishes the possibility of Poisson models, so the suitable alternative model is the NBR model. Nevertheless, the existence of excess zero as shown in (Fig 2) directs towards the likelihood of the zero-inflated models. The Vuong test was used to decide whether a zero-inflated model is more suitable than a standard count model. The calculated result of Vuong z-statistic = −2.814, p-value = 0.00244 at 0.05 level of significance indicates in favor of the alternate hypothesis that the zero-inflated model is more appropriate than the standard count model for this dataset. Finally, the ZINBR and HNBR models were fitted to assess the number of under-five deaths using these seven covariates. After fitting each model, the final model was selected based on the corresponding values of log-likelihood, AIC, and BIC. The lowest AIC and BIC values alongside the maximum value of log-likelihood as illustrated in Table 2 suggest that the ZINBR model is the better model than HNBR to estimate the number of under-five mortality encountered by a mother throughout her lifetime based on the NDHS 2022, however, these results showing no substantial differences. Moreover, the overall goodness of fit was evaluated by computing Pearson Chi-square statistic, which provided a value of 7800.16 with a p-value of 0.9909 at 8098 degrees of freedom. This result offers evidence in favor of the null hypothesis, indicating that the ZINBR model adequately fits the data.

**Table 2:** Comparison of different count models to predict the number of under-five mortality.

Selection criteria	Models		
	NBR	ZINBR	HNBR
Log-likelihood	−3959.72	−3944	−3946
AIC	7941.45	7929.47	7933.28
BIC	8018.47	8076.51	8080.32

**Fig 3:**
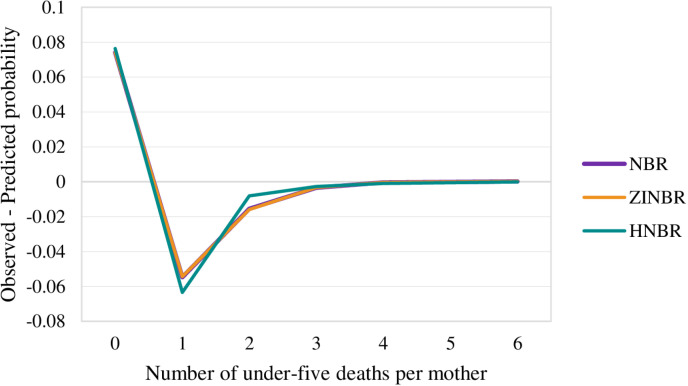
Plots of difference between observed and predicted probability of the number of under-five deaths for different models.

Fig 3 illustrates the curves for the difference between observed and predictive probabilities derived by fitting three count models related to the number of under-five mortality. Despite the visual similarities among the fitted curves, examination of the log-likelihood, AIC, and BIC values in Table 2 leads to the conclusion that the ZINBR model appears as the most suitable choice among the considered count models.

#### 3.2.3 Factors linked with the number of under-five mortality using the ZINBR model.

The fitted ZINBR model consists of two parts. First is the count model (negative binomial with log link), having a similar interpretation as negative binomial regression. The second part is the zero-inflation model (binomial with logit link). The estimates of this model are interpreted similarly to the standard logistic regression model. Table 3 shows the IRR, standard error, 95% confidence interval, z-values, and corresponding p-values for the various factors linked to the occurrence of under-five mortality. In the count model, the covariates including ecological region, mother smokes cigarettes, total CEB, source of drinking water, preceding birth interval, and birth order number were identified as statistically significant factors allied with the number of under-five mortality a mother has experienced throughout her lifetime, however, the place of residence of mothers whether they stayed in rural or urban areas and some levels of the covariates ecological region and source of drinking water remained insignificant at the 0.05 level of significance.

**Table 3:** Factors linked to the number of under-five mortality using the ZINBR model.

Count model coefficients (negative binomial with log link)							Zero-inflation model coefficients (binomial with logit link)					
Variables	IRR	Std. Error	95% CI for IRR		z value	p-value	AOR	Std. Error	95% CI for AOR		z value	p-value
Intercept	0.0927	0.0109	0.0736	0.1168	−9.933	<0.001	1.9542	1.2691	0.5472	6.9784	1.032	0.3022
**Ecological region**												
Mountain	1.1756	0.0913	1.0095	1.369	2.035	0.0418	1.7981	1.1685	0.503	6.4269	0.903	0.3666
Terai	1.0793	0.0922	0.9129	1.2761	0.84	0.4007	0.653	0.31	0.2575	1.6559	−0.898	0.3694
Hill (ref)												
**Place of residence**												
Rural	1.1038	0.0574	0.9967	1.2225	1.811	0.0701	0.4918	0.1847	0.2355	1.0268	−1.889	0.0588
Urban (ref)												
**Mother smokes cigarettes**												
Yes	1.2067	0.0919	1.0392	1.4011	2.453	0.0141	3.599	3.4534	0.5487	23.6026	1.335	0.182
No (ref)												
**Total CEB**												
3 or more	1.588	0.1549	1.3116	1.9226	2.105	0.0353	5.24E-08	7.3E-05	0.000	Inf	−0.012	0.9904
2 or less (ref)												
**Source of drinking water**												
Tube-well	0.7719	0.0868	0.6191	0.9623	−2.14	0.0323	0.7719	0.7294	0.2313	1.0268	−0.019	0.985
Piped water	0.9449	0.0862	0.7901	1.13	−0.578	0.5634	0.9449	0.9804	0.559	5.2591	0.943	0.3456
Others (ref)												
**Preceding birth interval**												
Less than 24 months	1.2061	0.0686	1.0788	1.3485	3.121	0.0018	0.1295	0.1063	0.0259	0.6474	−2.49	0.0128
24 months or more (ref)												
**Birth order number**												
4^th^ or above	3.5681	0.2261	3.1512	4.0401	20.082	<0.001	0.00192	11.43	0.000	Inf	0.000	0.9998
1^st^-3^rd^ (ref)												

As shown in Table 3, mothers from the mountain region as compared to the hill region has 1.176 (IRR: 1.1756, 95% CI: 1.0095–1.369) times higher incidence of experiencing under-five mortality, whereas those living in the terai region incidence rate for the number of under-five mortality increased by 1.079 (IRR: 1.0793, 95% CI: 0.9129–1.2761) times while holding the other covariates constant although it was not statistically significant. Likewise, the smoking habit of mothers has a substantial impact on the incidence rate of under-five mortality. The mothers who smoke cigarettes have a 1.207 (IRR: 1.2067, 95% CI: 1.0392–1.4011) times higher incidence of under-five mortality than those who did not smoke cigarettes. The fitted model also revealed that the incidence rate for the number of under-five mortality is positively and significantly related to the total CEB. The incidence of under-five deaths among mothers with a total CEB 3 or more is expected to have 1.59 (IRR: 1.588, 95% CI: 1.3116–1.9226) times greater than those having a total CEB 2 or less, while holding the other covariates constant. The source of drinking water has also emerged as a significant variable related to the under-five mortality. Those who drink water from tube-wells experienced approximately a 23% lower incidence of under-five deaths (IRR: 0.7719, 95% CI: 0.6191–0.9623) compared to those who drink water from other sources (like bottled water, Tanker truck, cart with small tank, etc.). Preceding birth interval is another covariate that shows a negative and significant correlation with the number of under-five deaths encountered by a mother during her lifetime. As compared to the preceding birth interval of 24 months or more, while holding the other variable constant in the model, the incidence of under-five deaths was 1.206 (IRR: 1.2061, 95% CI: 1.0788–1.3485) times higher among those mothers having a preceding birth interval less than 24 months. On the other hand, the incidence of experiencing under-five deaths was 3.568 (IRR: 3.5681, 95% CI: 3.1512–4.0401) times higher among those children with birth order 4^th^ or above than those with 1^st^- 3^rd^ birth order.

The second part of the ZINBR model in Table 3 shows the AOR, standard error, confidence interval for AOR, z-value, and p-value for the factor change in the odds of presence in the zero-count group (no under-five mortality) in comparison to non-zero count group (with at least one under-five mortality) in the zero-inflation model (binomial with logit link). Table 3 exhibits that the baseline odds of belonging in the zero-count group was 1.954. The preceding birth interval has a significant impact on the odds of being in the zero-count group, and it was the only significant covariate in the zero-inflation model. The odds of always being in the zero-count group for those children who had a preceding birth interval less than 24 months was 87% (AOR: 0.1295, 95% CI: 0.0259–0.6474) lower than those children with a birth interval 24 months or more. The variables ecological region, place of residence, mother smokes cigarettes, total CEB, source of drinking water, and birth order number were not statistically significant in this model. The broad confidence intervals observed for the AOR concerning the variables total CEB and birth order could potentially stem from convergence problems in the data.

## 4. Discussion

The primary purpose of this study was to elucidate the risk factors connected with the occurrence of under-five mortality in Nepal. This research investigated the incidence of under-five mortality among 14,821 mothers who had given birth at least once in their lifetime, utilizing the NDHS 2022 dataset. Among the total, 1280 mothers, accounting for 8.6%, encountered at least one under-five child mortality in their lifetime. The mean number of under-five mortality for each mother was calculated to be 0.11, with a standard deviation of 0.3924. To evaluate the factors correlated with the number of under-five mortality, different count models including PR, NBR, ZINBR, and HNBR were explored. Due to the presence of overdispersion and excess zeros, PR and NBR models were discarded from the study. By assessing the minimum AIC and BIC values and the maximum value of log-likelihood, it was concluded that the ZINBR model provided a better fit to the data in comparison to the NBR and HNBR models. We have observed that out of many factors included in the analysis, the ecological region, mother smokes cigarettes, total CEB, source of drinking water, preceding birth interval, and birth order number are significantly correlated with the number of under-five mortality.

The ecological region and source of drinking water are the contextual factors influencing under-five mortality. Additionally, a mother’s smoking habit is tied to her behavior or lifestyle, yet it can also reflect socio-economic influences [42]. The total CEB, the interval between previous births, and birth order are the demographic and health-related factors, though they may also carry some indirect socioeconomic implications. For instance, the total CEB per mother is influenced by factors such as the education of the mother, women empowerment, exposure to mass media, and wealth status of the household [43,44] whereas, the short interval between births may be affected by different socioeconomic aspects like the mother’s education, wealth status of the household, employment situation, the overall education level of the community, and living in rural areas [45,46]. Most of the factors identified in this study influencing under-five mortality appear to be directly or indirectly linked to a family’s socioeconomic status. Improving the socio-economic conditions of both individuals and the wider community, along with other circumstances, could serve as a stepping-stone in reducing the number of under-five mortality in Nepal. Moreover, the key predictors of under-five mortality appear to be interconnected and vary across different contexts, even though interaction effects of those predictors were not examined in this study. For example, the analysis of this data revealed a considerable link between maternal smoking and the household wealth index, suggesting that the effect of maternal smoking on under-five mortality differed by household wealth index. More specifically, mothers of those children who smoked and belong to lower-income households may possess a significantly higher risk of under-five mortality compared to those mothers from households having a higher wealth index. Similarly, the source of drinking water and total CEB are found to be associated with ecological regions. The risk of under-five mortality associated with the ecological region can be reduced by enhancing access to improved drinking water and raising awareness about the adverse effects of higher CEB on childhood mortality. The significant relationship between birth order and preceding birth interval obtained from the analysis may imply that a longer preceding birth interval may attenuate the risk associated with higher birth order. Future work may examine such interaction effects among the predictors so that such findings may be useful for adequate policy implications.

In 2022, Southern Asia had the third highest UFMR, behind sub-Saharan Africa and Oceania (excluding Australia and New Zealand). Compared to 1990, when the region recorded 127 deaths per 1,000 live births, Southern Asia saw a significant decline to 35 deaths per 1,000 live births in 2022. This rate was slightly lower than the global average of 37 deaths per 1,000 live births that year. Despite this, the Southern Asia sub-region contributed significantly to the global burden, accounting for 1.25 million of the 4.9 million under-five deaths worldwide, representing 26 percent of total global under-five mortality. Additionally, 63 percent of under-five deaths in this region occurred during the neonatal period. Over the past 26 years, Nepal has made notable strides in reducing under-five mortality. The UFMR in Nepal declined from 118 deaths per 1,000 live births in 1996 to33 in 2022, marking a greater decline compared to both the global and regional averages by 2022. However, the continued stagnation in neonatal mortality since 2016, along with a UFMR that remains above the SDG target of 25 deaths per 1,000 live births by 2030, indicates that Nepal needs to intensify its efforts to meet global health objectives [1,10].

Previous research in African countries has highlighted the mother’s region of residence, birth order, total CEB, birth interval, and source of drinking water as significant determinants of under-five mortality, consistent with the findings of this study. However, while maternal smoking appeared to be a significant factor in the context of Nepal, it was not observed as a contributing factor in the African region [47–58]. Similar to the findings of this study, research conducted in the European country Scotland, UK, as well as in various countries across the Western Pacific and Southeast Asia (including Cambodia, Indonesia, Lao People’s Democratic Republic, and Timor Leste), identified maternal smoking as a significant factor influencing the under-five mortality [59,60]. Similarly, two separate studies conducted in the Southeast Asian region observed the region of residence and previous birth interval as significant factors influencing under-five mortality, aligning with the results of this study [61,62]. Consistent with the findings of this study, research conducted in both the African and South Asian regions identified birth interval and birth order as significant factors affecting under-five mortality [63]. Furthermore, access to improved water facilities emerged as an important determinant of under-five mortality not only in Nepal, but also it was found significant in a systematic review covering all less-developed countries and in another study conducted across 70 low and middle-income countries [64,65].

The ecological region was one of the significant variables associated with the number of under-five mortality. Mothers from the mountain and terai regions experience a higher likelihood of the number of under-five mortality than those from the hill region. Poor standard of living, inaccessibility of easy and quality healthcare facilities due to poor road networks, lack of awareness about maternal and child health, etc. are the existing obstacles to reducing under-five mortality in the mountain and terai regions. Such a finding of regional disparity is consistent with other studies [47,48,61]. This result demands the proper allocation of resources in different regions based on evidence to curtail under-five mortality. The estimated result also showed that the smoking habit of mothers increases the risk of the number of under-five mortality, which is confirmed by the other studies [59,60,66]. The smoking habit of mothers is linked with birth defects, stillbirths, pre-term birth, and infant mortality. It has a devastating impact throughout childhood, and it sharply increases the risk of sudden infant death and birth defects. This study also revealed that the total CEB of mothers is positively connected with a greater risk of under-five mortality. As the number of children born to a mother increases by 3 or more, the child has a higher chance of death before attaining 5 years. This finding is congruent with the results obtained from previous studies [49,50]. The higher CEB indirectly increases childhood mortality by impacting maternal health, depleting household resources, and lowering the quality of care to children. To eliminate the effect of high CEB on under-five mortality, strategies should focus on promoting family planning and raising awareness among parents about the harmful effects of high CEB on maternal and child health.

Furthermore, the source of drinking water, whether they use water from the tube well or from other sources, was significantly correlated with the probability of experiencing under-five mortality. This result aligns with the findings of the earlier study, which observed that water treatment at the point of use and ensuring water quality are effective strategies for reducing diarrheal diseases, a major contributor to childhood mortality [64]. Besides, access to improved water also lowers the risk of mild or severe stunting and under-five mortality [51,65]. The preceding birth interval is another important variable related to under-five mortality. This study observed that the preceding birth interval of less than 24 months significantly increases the risk of under-five mortality than those with an interval of 24 months or more. Many studies are available to confirm this finding and advocate that shorter birth interval induces a higher probability of under-five mortality [52–54,58,62]. These findings emphasize that reproductive health interventions intended at reducing under-five mortality should concentrate on increasing the birth interval. The findings of this study also suggested that birth order number is an equally important determinant of under-five mortality. The birth order number was found to be positively related to the incidence of under-five mortality, and this result is similar to the findings of other studies [55–58]. Unlike the findings of this study, one study has observed that children with birth order three or less face a higher risk of death within the first five years than subsequent births [63]. The inconsistent findings among the different studies indicate the requirement for more extensive investigation.

## 5. Conclusions

The study aimed to determine the most promising factors associated with the number of under-five mortality a mother had experienced throughout her lifetime in Nepal using the NDHS 2022 dataset. It is concluded that 8.6% of mothers in Nepal experience at least one child death before attaining five years of age, with the average number of under-five mortality 0.11 per mother. The assessment of overdispersion and excess zeros in the dataset indicated in favor of the ZINBR and HNBR models. The ZINBR model was finally selected as an appropriate count model to fit the data by comparing the results of various methods.

The fitted ZINBR model explored that ecological region, mother smokes cigarettes, total CEB, source of drinking water, preceding birth interval, and the birth order number were significantly associated with the number of under-five mortality. Ecological regions exhibited significant variations in the number of under-five mortality. A higher incidence of under-five mortality was observed among the mothers who smoked cigarettes, and the total CEB was positively associated with under-five mortality. Moreover, the source of drinking water has been identified as an important covariate of under-five mortality. A preceding birth interval less than 24 months was strongly associated with an increased number of under-five mortality, whereas a significant positive relationship was obtained between birth order and the number of under-five mortality. Area-specific and evidence-based interventions specifically in the mountain and terai regions, and effective healthcare awareness programs to highlight the adverse effects of smoking habit, the importance of improved drinking water, the roles of birth interval, and birth order number are suggested to reduce the number of under-five mortality. This study recommends improving the existing healthcare services, particularly in marginalized areas, introducing effective education programs on child and maternal health, and elevating the overall quality of life as key measures to save newborn lives.

### 5.1 Strengths and limitations of the study

One strength of this study is its employment of the most comprehensive nationally representative NDHS 2022 data to identify and measure the effects of various factors related to under-five mortality in Nepal. The detailed analysis using descriptive and statistical models addresses the research gap in determining the factors associated with under-five mortality, especially in the context of Nepal. The conclusions drawn from this study, which utilizes a countrywide dataset, could benefit policymakers and concerned authorities in devising effective healthcare strategies aimed at the most at-risk groups to reduce childhood mortality. However, the assessment of under-five mortality was based on the available variables in the data and their suitability for analysis. Some variables, such as the duration of breastfeeding and gestational age of the child, were excluded due to computational complexity and a significant amount of missing values. The clinically significant causes of mortality under the age of five were not included in the study. Moreover, the NDHS 2022 utilized a women’s questionnaire to gather data from all eligible women aged 15–49. They were asked about various topics, including child mortality history, ANC, childbirth, PNC, working status, and their husbands’ background characteristics. Such information was obtained through the respondents’ self-reports, which could lead to potential recall errors, particularly over extended periods. For instance, older mothers may struggle to recall specific details about past events accurately due to memory lapses. They might not remember the exact birth date of their child, the age at which the child died, or whether the death occurred during the neonatal, infant, or under-five periods. This type of recall bias can introduce inaccuracies, including the underestimation or overestimation of under-five mortality rates, misreporting the timing of a child’s death, leading to biased age-specific mortality rates, and recalling recent or traumatic events more accurately than older or less significant ones. Our study did not incorporate several qualitative factors that could impact under-five mortality, such as awareness of childcare practices and hygiene, cultural beliefs and practices, and healthcare-seeking behaviors. Future research may focus on qualitative factors, such as exploring the importance of maternal participation in child healthcare decision-making, reforming detrimental social norms, building community trust in health systems, consciousness on childcare & hygiene practices, and examining environmental influences on child health. Similarly, it would be valuable to design a longitudinal study that assesses the effectiveness and sustainability of interventions at the household or community level aimed at reducing under-five mortality over time. Such studies may evaluate different interventions, including immunization programs, nutrition initiatives, sanitation and hygiene improvements, disease prevention and treatment efforts, strengthening health systems, and the effectiveness of awareness programs focused on maternal and child health.

## Supporting information

S1 File Code. Key R commands to fit the ZINBR model. (DOCX)

## Abbreviations

AIC: Akaike information criterion
ANC: Antenatal care
AOR: Adjusted odds ratio
ARR: Annual rate of reduction
BIC: Bayesian information criterion
CBS: Central bureau of statistics
CDRC: Central department research committee
CEB: Children ever born
CI: Confidence interval
DHS: Demographic and health survey
GLM: Generalized linear model
HNBR: Hurdle negative binomial regression
HPR: Hurdle Poisson regression
INGO: International non-governmental organization
IRR: Incidence rate ratio
LRT: Likelihood ratio test
LPG: Liquified petroleum gas
MDGs: Millennium development goals
NB: Negative binomial
NBR: Negative binomial regression
NDHS: Nepal demographic and health survey
NHRC: Nepal health research council
NPHC: , National population and housing census;
NSO: National statistics office
OLS: Ordinary least squares
PNC: Postnatal care
PR: Poisson regression
PSUs: Primary sampling units
SDGs: Sustainable development goals
TT: Tetanus toxoid
UFMR: Under-five mortality rate
UN: United nations
ZINB: Zero-inflated negative binomial
ZINBR: Zero-inflated negative binomial regression
ZIPR: Zero-inflated Poisson regression

## References

[1] Ministry of Health Nepal, New ERA, ICF. Nepal Demographic and Health Survey 2022. Kathmandu: Ministry of Health, Nepal; 2022. Available from: https://dhsprogram.com/publications/publication-FR379-DHS-Final-Reports.cfm

[2] United Nations Children’s Fund, UNICEF. Under-five mortality [internet]. 2021. Available from: https://data.unicef.org/topic/child-survival/under-five-mortality/

[3] Chao F, You D, Pedersen J, Hug L, Alkema L. National and regional under-5 mortality rate by economic status for low-income and middle-income countries: a systematic assessment. Lancet Glob Health. 2018;6(5):e535–47. doi: 10.1016/S2214-109X(18)30059-7 29653627 PMC5905403

[4] Yaya S, Bishwajit G, Okonofua F, Uthman OA. Under five mortality patterns and associated maternal risk factors in sub-Saharan Africa: A multi-country analysis. PLoS One. 2018;13(10):e0205977. doi: 10.1371/journal.pone.0205977 30359408 PMC6201907

[5] Mosley WH, Chen LC. An Analytical Framework for the Study of Child Survival in Developing Countries. Population and Development Review. 1984;10:25. doi: 10.2307/2807954 PMC2572391 12756980

[6] United Nations. Sustainable development goals. 2015. Available from: https://sdgs.un.org/publications/global-sustainable-development-report-2015-advance-unedited-version-gsdr-2015-17874

[7] United Nations. The Millennium Development Goals Report 2015. 2015. Available from: https://www.undp.org/publications/millennium-development-goals-report-2015

[8] Perin J, Mulick A, Yeung D, Villavicencio F, Lopez G, Strong KL, et al. Global, regional, and national causes of under-5 mortality in 2000-19: an updated systematic analysis with implications for the Sustainable Development Goals. Lancet Child Adolesc Health. 2022;6(2):106–15. doi: 10.1016/S2352-4642(21)00311-4 34800370 PMC8786667

[9] Azevedo JP, Banerjee A, Wilmoth J, Fu H, You D. Hard truths about under-5 mortality: call for urgent global action. Lancet. 2024;404(10452):506–8. doi: 10.1016/S0140-6736(24)00501-4 38492579

[10] United Nations Children’s Fund, UNICEF. Levels and trend of child mortality: Report 2023. Estimates developed by the UN Inter-agency Group for Child Mortality Estimation. 2024. Available from: https://data.unicef.org/resources/levels-and-trends-in-child-mortality/

[11] United Nations Children’s Fund, UNICEF. Children in South Asia. 2023. Available from: https://www.unicef.org/rosa/children-south-asia

[12] Verhulst A, Prieto JR, Alam N, Eilerts-Spinelli H, Erchick DJ, Gerland P, et al. Divergent age patterns of under-5 mortality in south Asia and sub-Saharan Africa: a modelling study. Lancet Glob Health. 2022;10(11):e1566–74. doi: 10.1016/S2214-109X(22)00337-0 36088913 PMC9588693

[13] National Planning Commission, Nepal. Nepal and the millennium development goals: final status report 2000-2015. 2016. Available from: https://www.npc.gov.np/images/category/MDG-Status-Report-2016_.pdf

[14] World Health Organization, Ministry of Health and Population, Nepal. Success factors for women’s and children’s health: Nepal. 2015. Available from: https://iris.who.int/handle/10665/254482

[15] Neupane S, Doku DT. Neonatal mortality in Nepal: a multilevel analysis of a nationally representative sample. [Corrected]. J Epidemiol Glob Health. 2014;4(3):213–22. doi: 10.1016/j.jegh.2014.02.001 25107657 PMC7333823

[16] Lamichhane R, Zhao Y, Paudel S, Adewuyi EO. Factors associated with infant mortality in Nepal: a comparative analysis of Nepal demographic and health surveys (NDHS) 2006 and 2011. BMC Public Health. 2017;17(1):53. doi: 10.1186/s12889-016-3922-z 28068969 PMC5223552

[17] Khadka KB, Lieberman LS, Giedraitis V, Bhatta L, Pandey G. The socio-economic determinants of infant mortality in Nepal: analysis of Nepal Demographic Health Survey, 2011. BMC Pediatr. 2015;15:152. doi: 10.1186/s12887-015-0468-7 26459356 PMC4603581

[18] Kc A, Jha AK, Shrestha MP, Zhou H, Gurung A, Thapa J, et al. Trends for Neonatal Deaths in Nepal (2001-2016) to Project Progress Towards the SDG Target in 2030, and Risk Factor Analyses to Focus Action. Matern Child Health J. 2019;24(Suppl 1):5–14. doi: 10.1007/s10995-019-02826-0 31773465 PMC7048722

[19] Joshi R, Sharma S, Teijlingen EV. Improving neonatal health in Nepal: major challenges to achieving millennium development goal 4. Health Sci J. 2013;7(3):247–57.

[20] Ghimire PR, Agho KE, Ezeh OK, Renzaho AMN, Dibley M, Raynes-Greenow C. Under-Five Mortality and Associated Factors: Evidence from the Nepal Demographic and Health Survey (2001-2016). Int J Environ Res Public Health. 2019;16(7):1241. doi: 10.3390/ijerph16071241 30965556 PMC6479835

[21] Dev R, Williams MF, Fitzpatrick AL, Connell FA. Topographical Differences of Infant Mortality in Nepal. Kathmandu Univ Med J (KUMJ). 2016;14(54):96–102. 28166062

[22] Paudel D, Shrestha IB, Siebeck M, Rehfuess EA. Neonatal health in Nepal: analysis of absolute and relative inequalities and impact of current efforts to reduce neonatal mortality. BMC Public Health. 2013;13:1239. doi: 10.1186/1471-2458-13-1239 24373558 PMC3890515

[23] Suwal JV. The main determinants of infant mortality in Nepal. Soc Sci Med. 2001;53(12):1667–81. doi: 10.1016/s0277-9536(00)00447-0 11762892

[24] Sreeramareddy CT, Harsha Kumar HN, Sathian B. Time trends and inequalities of under-five mortality in Nepal: a secondary data analysis of four demographic and health surveys between 1996 and 2011. PLoS One. 2013;8(11):e79818. doi: 10.1371/journal.pone.0079818 24224010 PMC3817106

[25] Cameron AC, Trivedi PK. Regression analysis of count data. 2nd ed. ed. Cambridge University Press; 1998.

[26] McCullagh P, Nelder J. Generalized linear models. 2nd ed. Chapman and Hall; 1989.

[27] Deb P, Norton EC. Modeling Health Care Expenditures and Use. Annu Rev Public Health. 2018;39:489–505. doi: 10.1146/annurev-publhealth-040617-013517 29328879

[28] Yang Z, Hardin JW, Addy CL. Score Tests for Zero-Inflation in Overdispersed Count Data. Communications in Statistics - Theory and Methods. 2010;39(11):2008–30. doi: 10.1080/03610920902948228

[29] Bhusal MK, Khanal SP. A Systematic Review of Factors Associated with Under-Five Child Mortality. Biomed Res Int. 2022;2022:1181409. doi: 10.1155/2022/1181409 36518629 PMC9744612

[30] Long JS. Regression models for categorical and limited dependent variables. Sage Publications; 1997.

[31] Hilbe J. Negative binomial regression. 2nd ed. Cambridge University Press; 2011.

[32] Lambert D. Zero-Inflated Poisson Regression, with an Application to Defects in Manufacturing. Technometrics. 1992;34(1):1. doi: 10.2307/1269547

[33] Bekalo DB, Kebede DT. Zero-Inflated Models for Count Data: An Application to Number of Antenatal Care Service Visits. Ann Data Sci. 2021;8(4):683–708. doi: 10.1007/s40745-021-00328-x

[34] Favero LP, Souza RDF, Belfiore P, Correa HL, Haddad MFC. Count data regression analysis: concepts, overdispersion detection, zero-inflation identification, and application with R. Practical Assess Res Eval. 2021;26(1):13. doi: 10.7275/44nn-cj68

[35] Zuur A, Leno E, Walker N, Saveliev A, Smith G. Zero truncated and zero-inflated models for count data in mixed effects models and extension in ecology with R. Springer; 2009.

[36] Mullahy J. Specification and testing of some modified count data models. Journal of Econometrics. 1986;33(3):341–65. doi: 10.1016/0304-4076(86)90002-3

[37] Gurmu S. Generalized hurdle count data regression models. Economics Letters. 1998;58(3):263–8. doi: 10.1016/s0165-1765(97)00295-4

[38] Cameron AC, Trivedi PK. Regression-based tests for overdispersion in the Poisson model. Journal of Econometrics. 1990;46(3):347–64. doi: 10.1016/0304-4076(90)90014-k

[39] Perumean-Chaney SE, Morgan C, McDowall D, Aban I. Zero-inflated and overdispersed: what’s one to do?. Journal of Statistical Computation and Simulation. 2012;83(9):1671–83. doi: 10.1080/00949655.2012.668550

[40] Vuong QH. Likelihood Ratio Tests for Model Selection and Non-Nested Hypotheses. Econometrica. 1989;57(2):307. doi: 10.2307/1912557

[41] Burnham KP, Anderson DR. Multimodel inference: Understanding AIC and BIC in model selection. Sociological Methods & Research. 2004; 33(2):261–304. doi: 10.1177/0049124104268644

[42] Gazmararian JA, Adams MM, Pamuk ER. Associations between measures of socioeconomic status and maternal health behavior. Am J Prev Med. 1996;12(2):108–15. doi: 10.1016/s0749-3797(18)30353-2 8777063

[43] Rahman A, Hossain Z, Rahman ML, Kabir E. Determinants of children ever born among ever-married women in Bangladesh: evidence from the Demographic and Health Survey 2017-2018. BMJ Open. 2022;12(6):e055223. doi: 10.1136/bmjopen-2021-055223 35768098 PMC9244679

[44] Adhikari R. Demographic, socio-economic, and cultural factors affecting fertility differentials in Nepal. BMC Pregnancy Childbirth. 2010;10:19. doi: 10.1186/1471-2393-10-19 20426863 PMC2885993

[45] Belachew TB, Asmamaw DB, Negash WD. Short birth interval and its predictors among reproductive age women in high fertility countries in sub-Saharan Africa: a multilevel analysis of recent Demographic and Health Surveys. BMC Pregnancy Childbirth. 2023;23(1):81. doi: 10.1186/s12884-023-05403-0 36717811 PMC9885595

[46] Pimentel J, Ansari U, Omer K, Gidado Y, Baba MC, Andersson N, et al. Factors associated with short birth interval in low- and middle-income countries: a systematic review. BMC Pregnancy Childbirth. 2020;20(1):156. doi: 10.1186/s12884-020-2852-z 32164598 PMC7069040

[47] Daniel K, Onyango NO, Sarguta RJ. A Spatial Survival Model for Risk Factors of Under-Five Child Mortality in Kenya. Int J Environ Res Public Health. 2021;19(1):399. doi: 10.3390/ijerph19010399 35010659 PMC8744899

[48] Gayawan E, Adarabioyo MI, Okewole DM, Fashoto SG, Ukaegbu JC. Geographical variations in infant and child mortality in West Africa: a geo-additive discrete-time survival modelling. Genus. 2016;72(1). doi: 10.1186/s41118-016-0009-8

[49] Ekholuenetale M, Wegbom AI, Tudeme G, Onikan A. Household factors associated with infant and under-five mortality in sub-Saharan Africa countries. ICEP. 2020;14(1). doi: 10.1186/s40723-020-00075-1

[50] Wegbom AI, Essi ID, Kiri VA. Survival analysis of under-five mortality and its associated determinants in Nigeria: evidence from a survey data. Int J Stat Appl. 2019;9(2):59–66.

[51] Gaffan N, Kpozehouen A, Degbey C, Ahanhanzo YG, Paraïso MN. Effects of household access to water, sanitation, and hygiene services on under-five mortality in Sub-Saharan Africa. Front Public Health. 2023;11:1136299. doi: 10.3389/fpubh.2023.1136299 37181724 PMC10173862

[52] Shifti DM, Chojenta C, Holliday E, Loxton D. Effects of short birth interval on neonatal, infant and under-five child mortality in Ethiopia: a nationally representative observational study using inverse probability of treatment weighting. BMJ Open. 2021;11(8):e047892. doi: 10.1136/bmjopen-2020-047892 34408041 PMC8375759

[53] Tesema GA, Worku MG, Alamneh TS, Teshale AB, Yeshaw Y, Alem AZ, et al. Estimating the impact of birth interval on under-five mortality in east african countries: a propensity score matching analysis. Arch Public Health. 2023;81(1):63. doi: 10.1186/s13690-023-01092-5 37085879 PMC10120214

[54] Budu E, Ahinkorah BO, Ameyaw EK, Seidu A-A, Zegeye B, Yaya S. Does Birth Interval Matter in Under-Five Mortality? Evidence from Demographic and Health Surveys from Eight Countries in West Africa. Biomed Res Int. 2021;2021:5516257. doi: 10.1155/2021/5516257 34055975 PMC8147536

[55] Debere HR, Adjiwanou V. The effects of reproductive variables on child mortality in Ethiopia: evidence from demographic and health surveys from 2000 to 2016. Reprod Health. 2024;21(1):4. doi: 10.1186/s12978-023-01734-5 38200569 PMC10777492

[56] Argawu AS, Mekebo GG. Zero-inflated Poisson regression analysis of factors associated with under-five mortality in Ethiopia using 2019 Ethiopian mini demographic and health survey data. PLoS One. 2023;18(11):e0291426. doi: 10.1371/journal.pone.0291426 37948385 PMC10637676

[57] Workie MS, Azene AG. Bayesian zero-inflated regression model with application to under-five child mortality. J Big Data. 2021;8(1). doi: 10.1186/s40537-020-00389-4

[58] Fenta SM, Fenta HM, Ayenew GM. The best statistical model to estimate predictors of under-five mortality in Ethiopia. J Big Data. 2020;7(1). doi: 10.1186/s40537-020-00339-0

[59] Andriani H, Putri S, Kosasih RI, Kuo H-W. Parental Smoking and Under-Five Child Mortality in Southeast Asia: Evidence from Demographic and Health Surveys. Int J Environ Res Public Health. 2019;16(23):4756. doi: 10.3390/ijerph16234756 31783665 PMC6926522

[60] World Health Organization (WHO). Tobacco control to improve child health and development: thematic brief. 2021. Available from: https://www.who.int/publications/i/item/9789240022218

[61] Woldeamanuel BT, Aga MA. Count Models Analysis of Factors Associated with Under-Five Mortality in Ethiopia. Glob Pediatr Health. 2021;8:2333794X21989538. doi: 10.1177/2333794X21989538 33623812 PMC7878955

[62] Islam MZ, Rahman Md M, Khan Md N. Effects of short birth interval on different forms of child mortality in Bangladesh: Application of propensity score matching technique with inverse probability of treatment weighting. PloS One. 2023;18(4):e0284776. doi: 10.1371/journal.pone.0284776 37083714 PMC10121045

[63] Amir-Ud-Din R, Naz L, Rubi A, Usman M, Ghimire U. Impact of high-risk fertility behaviours on underfive mortality in Asia and Africa: evidence from Demographic and Health Surveys. BMC Pregnancy Childbirth. 2021;21(1):344. doi: 10.1186/s12884-021-03780-y 33933011 PMC8088561

[64] Fewtrell L, Kaufmann RB, Kay D, Enanoria W, Haller L, Colford JM Jr. Water, sanitation, and hygiene interventions to reduce diarrhoea in less developed countries: a systematic review and meta-analysis. Lancet Infect Dis. 2005;5(1):42–52. doi: 10.1016/S1473-3099(04)01253-8 15620560

[65] Fink G, Günther I, Hill K. The effect of water and sanitation on child health: evidence from the demographic and health surveys 1986-2007. Int J Epidemiol. 2011;40(5):1196–204. doi: 10.1093/ije/dyr102 21724576

[66] Lawder R, Whyte B, Wood R, Fischbacher C, Tappin DM. Impact of maternal smoking on early childhood health: a retrospective cohort linked dataset analysis of 697 003 children born in Scotland 1997-2009. BMJ Open. 2019;9(3):e023213. doi: 10.1136/bmjopen-2018-023213 30898797 PMC6475204

