# Supplementary material for: Statistical models for predicting the number of under-five mortality in Nepal
Source: PLoS One. 2025 May 21;20(5):e0324321. doi: 10.1371/journal.pone.0324321 (PMC12094737; doi:10.1371/journal.pone.0324321)
Supplement: S1 File — (DOCX) [file pone.0324321.s001.docx]

**Key R commands to fit the ZINBR Model**

**Install the following packages for analysis (R version 4.3.2)**

install.packages("MASS")

install.packages("pscl")

install.packages("AER")

install.packages("foreign")

install.packages("lmtest")

install.packages("rms")

install.packages("msm")

**Fit the ZINBR model**

zinb<- zeroinfl(No._underfive_deaths ~ Ecological_region + Total_CEB + Preceding_BI +

Birth_order_no. + Place_residence + Sdrinking_water +

Msmokes_cigarettes|Ecological_region + Total_CEB + Preceding_BI +

Birth_order_no. + Place_residence + Sdrinking_water +

Msmokes_cigarettes, link = "logit", dist = "negbin", data = countdata)

summary(zinb)

**Calculate IRRs, standard error and confidence intervals for the count part of the model**

coefficients<- coef(zinb)

IRRs<-exp(coefficients)

**Standard errors for IRRs using delta method**

standard_errors<- summary(zinb)$coef[, "Std. Error"]

SE_IRRs<- IRRs*standard_errors

**Compute confidence intervals for IRRs**

log_CI<- cbind(coefficients-1.96*standard_errors,

coefficients+1.96*standard_errors)

**Confidence intervals for IRRs**

CI_IRRs<- exp(log_CI)

**Print IRRs, standard errors, and confidence intervals**

results<- cbind(IRRs, SE_IRRs, CI_IRRs)

print(results)

**Calculation of AIC and BIC of ZINBR model**

AIC(zinb)

BIC(zinb)

**Calculation of AOR, standard errors, and confidence interval for the zero-inflation part of the model**

**Extract coefficients and standard errors**

coef_ <- coef(zinb)

std_errors <- sqrt(diag(vcov(zinb)))

**Calculation of Adjusted Odds Ratios (AOR)**

aor <- exp(coef_)

**Delta method for standard errors of the AOR**

std_error_aor <- aor * std_errors # Because AOR = exp(coef)

**Compute confidence intervals for coefficients and AOR**

conf_int <- confint(zinb, level = 0.95)

conf_int_aor <- exp(conf_int) # Exponentiate to get confidence intervals for AOR

**Data frame with results**

results <- data.frame(

Component = c("Count Model", "Zero-Inflation Model"),

Coefficients = coef_,

Std_Errors = std_errors,

AOR = aor,

Std_Error_AOR = std_error_aor,

Conf_Lower_AOR = conf_int_aor[, 1], # Lower bound of AOR CI

Conf_Upper_AOR = conf_int_aor[, 2] # Upper bound of AOR CI

print(results)

**Pearson Chi-square statistic for overall goodness of fit**

pearson_chi <- sum(residuals(zinb, type = "pearson")^2)

df_pearson <- sum(zinb$df.residual)

p_value_pearson <- 1 - pchisq(pearson_chi, df_pearson)

cat("Pearson Chi-square:", pearson_chi, "with", df_pearson, "degrees of freedom\n")

**Output the results**

cat("Pearson Chi-square:", pearson_chi, "\n")

cat("P-value for Pearson Chi-square:", p_value_pearson, "\n")
